# Characterization of autonomic symptom burden in long COVID: A global survey of 2,314 adults

**DOI:** 10.3389/fneur.2022.1012668

**Published:** 2022-10-19

**Authors:** Nicholas W. Larsen, Lauren E. Stiles, Ruba Shaik, Logan Schneider, Srikanth Muppidi, Cheuk To Tsui, Linda N. Geng, Hector Bonilla, Mitchell G. Miglis

**Affiliations:** ^1^Department of Neurology and Neurological Sciences, Stanford University, Palo Alto, CA, United States; ^2^Department of Neurology, Stony Brook University Renaissance School of Medicine, Stony Brook, NY, United States; ^3^Dysautonomia International, East Moriches, NY, United States; ^4^Stanford Sleep Center, Department of Psychiatry and Behavioral Sciences Stanford University, Redwood City, CA, United States; ^5^Department of Statistics, University of Chicago, Chicago, IL, United States; ^6^Department of Medicine, Stanford University, Palo Alto, CA, United States

**Keywords:** post-acute sequelae of COVID-19, post-acute COVID, long COVID, dysautonomia, autonomic, autonomic dysfunction

## Abstract

**Background:**

Autonomic dysfunction is a known complication of post-acute sequelae of SARS-CoV-2 (PASC)/long COVID, however prevalence and severity are unknown.

**Objective:**

To assess the frequency, severity, and risk factors of autonomic dysfunction in PASC, and to determine whether severity of acute SARS-CoV-2 infection is associated with severity of autonomic dysfunction.

**Design:**

Cross-sectional online survey of adults with PASC recruited through long COVID support groups between October 2020 and August 2021.

**Participants:**

2,413 adults ages 18–64 years with PASC including patients who had a confirmed positive test for COVID-19 (test-confirmed) and participants who were diagnosed with COVID-19 based on clinical symptoms alone.

**Main measures:**

The main outcome measure was the Composite Autonomic Symptom 31 (COMPASS-31) total score, used to assess global autonomic dysfunction. Test-confirmed hospitalized vs. test-confirmed non-hospitalized participants were compared to determine if the severity of acute SARS-CoV-2 infection was associated with the severity autonomic dysfunction.

**Key results:**

Sixty-six percent of PASC patients had a COMPASS-31 score >20, suggestive of moderate to severe autonomic dysfunction. COMPASS-31 scores did not differ between test-confirmed hospitalized and test-confirmed non-hospitalized participants [28.95 (15.62, 46.60) vs. 26.4 (13.75, 42.10); *p* = 0.06].

**Conclusions:**

Evidence of moderate to severe autonomic dysfunction was seen in 66% of PASC patients in our study, independent of hospitalization status, suggesting that autonomic dysfunction is highly prevalent in the PASC population and independent of the severity of acute COVID-19 illness.

## Introduction

A substantial number of those with coronavirus disease 19 (COVID-19) will experience long-lasting symptoms (1, 2), a condition referred to as post-acute sequelae of SARS-CoV-2 (PASC), or long COVID. Although PASC is not universally defined, it is often identified as symptoms of COVID-19 lasting at least 30 days (3). PASC may include a diverse constellation of symptoms, many of which appear to be autonomic in nature (4). In fact, many of the symptoms of PASC have been documented in individuals with autonomic disorders prior to the COVID-19 pandemic (4), suggesting either a shared pathophysiological mechanism or unmasking of a pre-existing subclinical condition. The possibility of a shared pathophysiological mechanism has prompted our interest in PASC as a framework for better understanding post-viral dysautonomia. This is especially important given the significant level of disability experienced by those with autonomic disorders.

While there have been online surveys of patients with PASC (5), none have specifically addressed the prevalence of autonomic dysfunction and how autonomic symptom burden is associated with other features of the illness. In this context, we designed and conducted a global online survey of individuals with PASC with the goal of assessing the frequency and severity of autonomic symptoms in this population. We also aimed to evaluate which pre-existing conditions are associated with an increased risk of developing autonomic dysfunction in PASC, and to determine whether the severity of acute COVID-19 illness is associated with more severe autonomic dysfunction in PASC.

## Materials and methods

### Survey administration

An online survey study was administered to adults ≥18 years of age who had self-reported, clinician-diagnosed or test-confirmed SARS-CoV-2 infection and symptoms persisting beyond 30 days. The study was approved by the Stanford University and Stony Brook University Institutional Review Boards, and all participants gave digital informed consent before starting the survey. The participants were recruited through PASC support groups and social media channels between October 2020 and August 2021. To avoid potential selection bias, study advertisements were placed in Long COVID and acute COVID social media support groups and websites with a reach to large numbers of acute and post-acute COVID patients such as Survivor Corps (191K members), COVID-19 Long Haulers Advocacy Project (8.4K members), and Long Haul COVID Fighters (6.3K members). Study recruitment language was neutral and did not mention dysautonomia. Self-suspected and clinician-diagnosed COVID-19 was categorized as “test-unconfirmed” and polymerase chain reaction (PCR), antigen and antibody (if unvaccinated)-confirmed COVID-19 was categorized as “test-confirmed” for analytic purposes. All participants completed an online, English-language survey consisting of demographic information, medical history, severity of acute COVID-19 illness, and a series of validated-questionnaires. Data were collected *via* the online Research Electronic Data Capture (REDCap) platform.

The COMPASS-31 is a widely-utilized patient questionnaire that provides a quantitative assessment of the severity and distribution of autonomic symptoms (6). This questionnaire generates a weighted score from 0 to 100, and questions fall into one of six domains: orthostatic intolerance, vasomotor, secretomotor, gastrointestinal, bladder, and pupillomotor function. A COMPASS-31 score of ≥20 suggests moderate-to-severe autonomic dysfunction (7). The Orthostatic Hypotension Questionnaire (OHQ) is used to evaluate the severity of orthostatic intolerance and the functional impact of neurogenic orthostatic hypotension (8), and, by extension, other disorders of orthostatic intolerance including POTS. Scores range from 0 to 100, with higher scores representing higher symptom burden. The Fatigue Severity Scale (FSS) is a 9-item instrument to assess the effect that fatigue has on daily functioning (9). Scores range between 9-63 points, with a score of 36 or more suggesting abnormal levels of fatigue. The Epworth Sleepiness Scale (ESS) is a 24-point scale that quantifies the likelihood of dozing in various situations over the preceding weeks. Scores >10 suggest excessive daytime sleepiness (10). The Neuropathic Pain Scale (NPS) scale measures 10 specific qualities associated with neuropathic pain. Scores range from 0 to 100, with higher scores suggesting greater disability (11). The Generalized Anxiety Disorder Assessment (GAD-7) is a valid and efficient tool to screen for generalized anxiety disorder (12). The scores range from 0 to 24 with the cut-offs of 5, 10, and 15 representing mild, moderate, and severe levels of anxiety. The RAND 36-Item Health Survey (RAND-36) is a quality of life measure that incorporates eight health concepts including physical functioning, bodily pain, role limitations due to physical health problems, role limitations due to personal or emotional problems, emotional well-being, social functioning, energy/fatigue, and general health perceptions (13). Each health concept is scored on a 0–100 range, with lower scores representing greater disability.

### Exclusion criteria

Participants were removed from the dataset based on the following criteria: incomplete dataset, symptom duration < 30 days, symptom onset before November 2019 and age ≥65 years. Participants ≥65 years of age were excluded due to concern of survivor bias secondary to disproportionately high mortality in this age range.

### Statistical analysis

A tiered approach to analyses was used to first determine comparability of the test-confirmed and test-unconfirmed groups, with subsequent analyses exploring the associations with clinical severity (i.e., hospitalized vs. non-hospitalized) being assessed only in the diagnostically confirmed group. Categorical variables are presented as count and percentages, and continuous variables as mean±SD for Gaussian variables or median and interquartile range for non-Gaussian variables, as confirmed by the Shapiro-Wilk test for normality. For Gaussian variables, comparisons were performed using *t*-tests, with Cohen's *d* for effect size estimation. For non-Gaussian variables, comparisons were performed with the Wilcoxon rank sum test, with *r* representing effect sizes. χ^2^ or Fisher's exact test (when counts fell below 5 in any category) were used to compare categorical variables between groups, and Cramér's *V* provided an estimation of effect size. Spearman correlation coefficients were calculated to assess strength and alignment of associations between COMPASS-31 scores and other survey measures. A correlation coefficient (r) of >0.5 or < -0.5 was interpreted as a meaningful correlation. A statistical threshold of α = 0.05 was set and Bonferroni correction for multiple comparisons was performed for each major analysis. All methods were implemented in python v3.7.3 (14).

## Results

### Demographics

**Figure 1:**
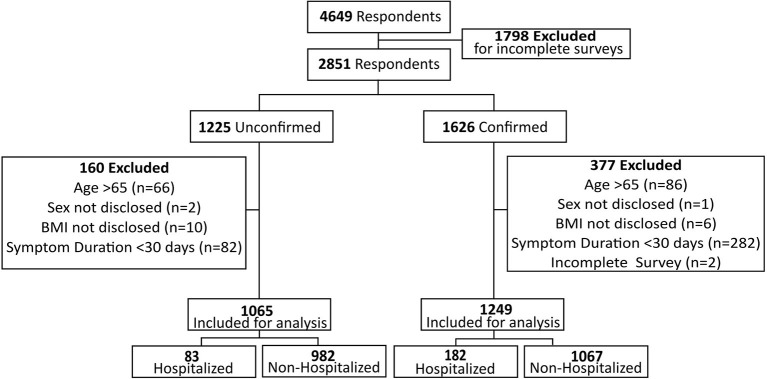
Participant flow diagram.

In total, 4,649 participants responded to the survey (Figure 1). Ultimately, 2,314 survey responses were analyzed from 34 different countries, including 1,249 test-confirmed participants and 1,065 test-unconfirmed participants. Most participants resided in the USA (77.6%), were female (87.3%), white (89.5%), and between the ages of 31 and 65 years (86.7%) (Table 1). The test-unconfirmed group had a longer duration of symptoms, with 82.4% of participants noting symptom onset ≥6 months, compared to 37.2% in the test-confirmed group. Most participants were never hospitalized for their acute infection (88.6%), < 2% were admitted to the ICU, and < 1% were intubated.

**Table 1:** Demographics of PASC participants.

**Variable**	**Test confirmed** **(*N =* 1,249, %)**	**Test unconfirmed** **(*N =* 1,065, %)**
Female	1,079 (86.4%)	941 (88.4%)
Male	170 (13.6%)	124 (11.6%)
**Age (years)**		
Age 18–30	182 (14.6%)	126 (11.8%)
Age 31–45	494 (39.6%)	457 (43%)
Age 46–65	573 (45.9%)	482 (45.3%)
Race/Ethnicity*		
White	1,099 (88%)	972 (91.3%)
Hispanic	69 (5.5%)	36 (3.4%)
Asian	42 (3.4%)	43 (4%)
Native American/Indian	28 (2.2%)	27 (2.5%)
Black	28 (2.4%)	14 (1.3%)
Middle Eastern	11 (1 %)	12 (1.1%)
Hawaiian/Pacific Islander	6 (0.5%)	1 (0.1%)
Biracial/Multiracial	35 (2.8%)	36 (3.4%)
Not disclosed/unknown	17(1.4%)	17 (1.6%)
Average BMI**		
Underweight (< 18.5)	41 (3.3%)	60 (5.6%)
Normal (18.5–24.9)	421 (33.7%)	484 (45.4%)
Overweight (25–29.9)	316 (25.3%)	277 (26%)
Obese (30 and above)	456 (36.6%)	228 (21.4%)
**Time from symptom onset to survey completion**		
30 days−3 months	426 (34.1%)	49 (4.6%)
3–6 months	359 (28.7%)	138 (13%)
6–9 months	352 (28.2%)	584 (54.8%)
9 months and greater	112 (9%)	294 (27.6%)
**Testing Status**		
Self-suspected (no test)	0	388 (36.4%)
Physician diagnosed (no test)	0	677 (63.6%)
Symptoms only	0	1,065 (100%)
Diagnostic (RT-PCR/antigen)	899 (72%)	0
Antibody (IgG, IgM or both)	143 (11.4%)	0
Diagnostic and antibody	207 (16.6%)	0
**COVID Severity**		
No hospital admission	1,069 (86%)	982 (92.2%)
Hospital admission	180 (14.4%)	83 (7.8%)
ICU admission	41 (3.3 %)	5 (0.5%)
Intubated	12 (1%)	2 (0.2%)
Supplemental oxygen	134 (10.7%)	46 (4.3%)
Countries of residence***		
USA	1,069 (85.6%)	727 (68.3%)
United Kingdom	26 (2.1%)	128 (12%)
Canada	28 (2.2%)	103 (9.7%)
Germany	27 (2.2%)	10 (0.9%)
Sweden	6 (0.5%)	17 (1.6%)

*Some respondents checked more than 1 category, **Some data was incomplete (N = 1,234 testing confirmed, N = 1,049 testing unconfirmed), ***Participants from 34 countries responded to the survey, only countries with a respondence rate of 1% of greater are listed.

### Medical history

The most reported pre-existing conditions were anxiety, depression, prior history of smoking or vaping, vitamin D deficiency, asthma, food or environmental allergies, obesity, hypertension, and a history of autoimmune disease (Table 2). A pre-existing diagnosis of autonomic dysfunction was reported by 5.1% of test-confirmed and 8.3% of test-unconfirmed participants, with POTS being the most commonly reported autonomic disorder, followed by neurally-mediated syncope, orthostatic intolerance, orthostatic hypotension (OH), inappropriate sinus tachycardia, and autonomic neuropathy.

**Table 2:** Medical history of survey respondents prior to COVID-19.

**Variable**	**Test Confirmed** **(*N =* 1,249, %)**	**Test Unconfirmed** **(*N =* 1,065, %)**
Anxiety	412 (33%)	252 (23.7%)
Depression	299 (23.9%)	225 (21.1%)
Former smoker/vaper	255 (20.4%)	250 (23.5%)
Vitamin D deficiency	258 (20.7%)	201 (18.9%)
Asthma	256 (20.5%)	203 (19.1%)
Food or environmental allergies	195 (15.6%)	196 (18.4%)
Obesity	254 (20.3%)	118 (11.1%)
High blood pressure	212 (17%)	109 (10.2%)
Autoimmune disease	136 (10.9%)	128 (12%)
Insomnia	103 (8.2%)	91 (8.5%)
Anemia	112 (9%)	79 (7.4%)
Current smoker/vaper	68 (5.4%)	73 (6.9%)
Obstructive sleep apnea	86 (6.9%)	54 (5.1%)
Neuropathy	50 (4%)	59 (5.5%)
Ehlers-Danlos syndrome	34 (2.72%)	67 (6.3%)
Diabetes	52 (4.2%)	29 (2.7%)
Cancer	37 (3%)	24 (2.3%)
Former chewing tobacco user	10 (0.8%)	7 (0.7%)
Current chewing tobacco user	6 (0.5%)	4 (0.4%)
Any form of dysautonomia	64 (5.1%)	88 (8.3%)
Postural orthostatic tachycardia syndrome	51 (4.1%)	73 (6.8%)
Vasovagal or neurocardiogenic syncope	15 (1.2%)	25 (2.3%)
Orthostatic intolerance	9 (0.7%)	17 (1.6%)
Orthostatic hypotension	7 (0.6%)	17 (1.6%)
Inappropriate sinus tachycardia	9 (0.7%)	13 (1.2%)
Autonomic neuropathy	5 (0.4%)	11 (1%)
Autonomic failure	2 (0.2%)	4 (0.4%)
Autoimmune autonomic ganglionopathy	2 (0.2%)	0
Other types of dysautonomia	3 (0.2%)	6 (0.6%)

### PASC symptom prevalence

**Figure 2:**
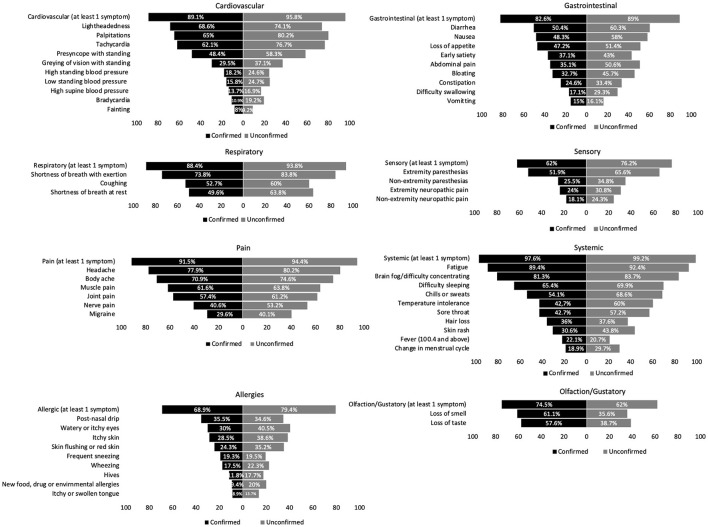
Prevalence of PASC symptoms.

We grouped 53 PASC symptoms into eight domains: respiratory, cardiovascular, gastrointestinal, pain, allergic, sensory, smell, and systemic (Figure 2). The most reported symptoms were fatigue, brain fog, headache, shortness of breath with exertion, body aches, palpitations, lightheadedness, tachycardia, and difficulty sleeping. Loss of taste and loss of smell were also commonly reported.

### Relationship of pre-existing conditions with autonomic symptom burden

All pre-existing conditions were associated with greater autonomic symptom burden as measured by COMPASS-31 total scores, except for high blood pressure (both groups), obesity (test-confirmed group only), and former smoker/vaper (test-unconfirmed group only) (Table 3). Participants with a history of asthma, obesity (test-unconfirmed group only), vitamin D deficiency, autoimmune disease, food or environmental allergies, anxiety, depression, and smoking/vaping (test-confirmed group only) had significantly higher COMPASS-31 scores than those who did not report these conditions. These associations were true in both test-confirmed and test-unconfirmed cohorts, unless otherwise noted.

**Table 3:** Impact of medical conditions on autonomic symptom burden.

	**Reported** ***(N, Mean ±SD)***	**Not Reported** ***(N, Mean ±SD)***	***T*-Test Stat**	**Effect Size**	***P*-value**	**df**
**Pre-existing conditions: test confirmed patients**						
High blood pressure	212, 29.0 ± 18.5	1,037, 29.2 ± 17.8	0.1	−0.01	0.92	1,247
Asthma	256, 33.1 ± 18.3	993, 28.1 ± 17.7	−4.0	0.28	*p < * 0.01	1,247
Obesity	254, 31.6 ± 17.8	995, 28.5 ± 18.0	−2.48	0.17	0.01	1,247
Vitamin D deficiency	258, 34.1 ± 17.7	991, 27.9 ± 17.8	−5.0	0.35	*p < * 0.01	1,247
Autoimmune disease	136, 35.7 ± 18.2	1,113, 28.4 ± 17.8	−4.51	0.41	*p < * 0.01	1,247
Food or environmental allergies	195, 34.2 ± 18.1	1,054, 28.2 ± 17.8	−4.31	0.33	*p < * 0.01	1,247
Anxiety	412, 34.2± 17.3	837, 26.7 ± 17.8	−7.10	0.43	*p < * 0.01	1,247
Depression	299, 34.6 ± 17.4	950, 27.4 ± 17.8	−6.06	0.41	*p < * 0.01	1,247
Smoker/Vaper	321, 33.2 ± 18.5	928, 27.8 ± 17.6	−4.71	0.3	*p < * 0.01	1,247
**Pre-existing conditions: test unconfirmed patients**						
High blood Pressure	109, 36.2 ± 18.9	956, 33.5 ± 17.3	−1.51	0.15	0.13	1,063
Asthma	203, 37.5 ± 17.2	862, 32.9 ± 17.4	−3.38	0.26	*p < * 0.01	1,063
Obesity	118, 38.3 ± 18.3	947, 33.2 ± 17.3	−3.03	0.29	*p < * 0.01	1,063
Vitamin D deficiency	201, 38.4 ± 16.7	864, 32.7 ± 17.4	−4.24	0.32	*p < * 0.01	1,063
Autoimmune disease	128, 38.7 ± 17.3	937, 33.1 ± 17.4	−3.46	0.31	*p < * 0.01	1,063
Food or environmental allergies	196, 39.1 ± 17.4	869, 32.6 ± 17.2	−4.80	0.36	*p < * 0.01	1,063
Anxiety	252, 39.7 ± 17.1	813, 31.9 ± 17.1	−6.28	0.44	*p < * 0.01	1,063
Depression	225, 39.2 ± 17.1	840, 32.3 ± 17.3	−5.34	0.39	*p < * 0.01	1,063
Smoker/Vaper	321, 34.7 ± 16.9	744.33.4 ± 17.7	−1.18	0.07	0.24	1,063

### Test-confirmed vs. test-unconfirmed participants

Sixty-six percent of PASC participants had a median COMPASS-31 score of 20 or greater, suggestive of moderate to severe autonomic dysfunction. In order to prevent selection bias from influencing these results, we excluded patients self-reporting a history of autonomic dysfunction from this calculation. Test-unconfirmed participants had significantly higher COMPASS-31 scores than test-confirmed participants (26.8 ± 28.9 vs. 34.7 ± 27.8; *p* < 0.0018) as well as significantly higher scores in all COMPASS-31 subdomains (Table 4) except for secretomotor (4.29 ± 6.43 vs. 4.29 ± 8.57; *p* = 0.05). Test-confirmed participants had a higher BMI than test-unconfirmed participants (27.1 ± 10.49 vs. 24.8 ± 7.19; *p* < 0.0018), while test-unconfirmed participants were more likely to report longer symptom duration (137 ± 136 days vs. 217 ± 84 days; *p* < 0.0018). Both test-confirmed and test-unconfirmed participants had elevated OHQ scores [55 (35,73) vs. 55 (40,70)], elevated FSS scores [54 (41,61) vs. 56 (41,62)] and mildly elevated GAD-7 scores [8 (5, 14) vs. 7 (4, 12)]. The test-unconfirmed group had higher FSS (54 ± 20 vs. 56 ± 16; *p* < 0.0018) and NPS (12 ± 42 vs. 22 ± 46; *p* < 0.0018) scores than the test-confirmed group, while the test-confirmed group had elevated GAD-7 scores (8 ± 9 vs. 7 ± 8, *P* < 0.0018). Neither group had ESS scores suggestive of excessive daytime sleepiness [8 (4, 13) vs. 8 (4, 12)]. Both test-confirmed and test-unconfirmed participants had relatively low scores across all RAND subdomains (Table 4). The test-unconfirmed group had lower scores on the RAND-36 subdomains of physical functioning (38.89 ± 50 vs. 22 ± 46; *P* < 0.0018), role limitations due to physical health (0 ± 25 vs. 0 ± 0; *P* < 0.0018), social functioning (37.5 ± 37.5 vs. 25 ± 37.5; *P* < 0.0018), and general health (40 ± 35 vs. 35 ± 25; *P* < 0.0018).

**Table 4:** Comparison of demographic variables and symptom scores in test-confirmed and test-unconfirmed participants.

**Variables**	**Test confirmed** **(*N =* 1,249)** ***Median, IQR*** ***[25th, 75th]***	**Test unconfirmed(*N =* 1,065)** ***Median, IQR*** ***[25th, 75th]***	**Test statistic**	**Effect size**	***p*-value**	**Comparison**
Age	44,17 [36, 53]	44,16 [36, 52]	−0.46	−0.01	0.65	N/A
BMI	27.1,10.49 [22.89, 33.38]	24.8,7.19 [21.92, 29.12]	7.99	0.17	*p < * 0.0018	C>U
Sex M:F (%F)	170:1079 (86%)	124:941 (88%)	1.83	0.03	0.18	N/A
Symptom Duration	137,136.00 [68, 204]	217,84 [191, 275]	−22.2	−0.46	*p < * 0.0018	U>C
Total COMPASS-31 Score	26.8,28.9 [13.9, 42.8]	34.7,27.8 [19.2, 47.0]	−6.38	−0.13	*p < * 0.0018	U>C
**Compass-31 Subcategories**						
Orthostatic Intolerance	16,24 [0, 24]	16,16 [8, 24]	−5.32	−0.11	*p < * 0.0018	U>C
Vasomotor	0,0 [0, 0]	0,1.67 [0, 1.67]	−6	−0.12	*p < * 0.0018	U>C
Secretomotor	4.29,6.43 [0, 6.43]	4.29,8.57 [0, 8.57]	−2	−0.04	0.05	N/A
Gastrointestinal	8.04,6.25 [4.46, 10.72]	8.93,6.25 [5.36, 11.61]	−4.05	−0.08	*p < * 0.0018	U>C
Bladder	0,2.22 [0, 2.22]	1.11,2.22 [0, 2.22]	−3.39	−0.07	*p < * 0.0018	U>C
Pupillomotor	2,2 [1, 3]	2.33,1.67 [1.33, 3.0]	−4.36	−0.09	*p < * 0.0018	U>C
Orthostatic Hypotension Questionnaire	55,38 [35, 73]	55,30 [40, 70]	−0.92	−0.02	0.36	N/A
Fatigue Severity Scale	54,20 [41, 61]	56,16 [41, 62]	−4.08	−0.08	*p < * 0.0018	U>C
Epworth Sleepiness Scale	8,9 [4, 13]	8,8 [4, 12]	2.92	0.06	0.003	N/A
GAD-7 Questionnaire	8,9 [5, 14]	7,8 [4, 12]	5.24	0.11	*p < * 0.0018	C>U
Neuropathic Pain Scale	12,42 [0, 42]	22,46 [0, 46]	−3.56	−0.07	*p < * 0.0018	U>C
**Rand-36 Subcategories**						
Physical function	38.89,50 [16.67, 66.67]	22.0, 46 [0, 46]	14.53	0.30	*p < * 0.0018	C>U
Role limitations due to physical health	0,25 [0, 25]	0,0 [0, 0]	4.58	0.10	*p < * 0.0018	C>U
Role limitations due to emotional problems	33.33,100 [0, 100]	33.33,100 [0, 100]	−2.05	−0.04	0.04	N/A
Energy/Fatigue	20,30 [5, 35]	20,25 [5, 30]	2.25	0.05	0.02	N/A
Emotional well-being	55,35 [35, 70]	60,30 [40, 70]	−2.89	−0.06	0.004	N/A
Social functioning	37.5,37.5 [12.5, 50]	25,37.5 [12.5, 50]	3.69	0.08	*p < * 0.0018	C>U
Pain	45,45 [22.5, 67.5]	45,35 [32.5, 67.5]	1.41	0.03	0.16	N/A
General health	40,35 [25, 60]	35,25 [25, 50]	7.3	0.15	*p < * 0.0018	C>U

Finally, to ensure that the inclusion of participants with a history of autonomic dysfunction did not influence total COMPASS-31 scores, we conducted a *post-hoc* analysis after excluding these participants (64 patients in the test-confirmed group and 88 patients in the test-unconfirmed group). We found no change in our results as the difference between groups remained significant [25.7 (13.6, 41.7) vs. 32.7 (18, 44.9), *p* < 0.0018, significance threshold *p* < 0.0018].

### Test confirmed hospitalized vs. non-hospitalized participants

To further evaluate the association of disease severity with severity of autonomic dysfunction in PASC, we analyzed only the test-confirmed subset of participants (Table 5). Both test-confirmed hospitalized and non-hospitalized participants had median COMPASS-31 scores >20 [hospitalized 28.95 (15.62,46.60) vs. non-hospitalized 26.4 (13.75,42.10)] suggesting moderate-to-severe autonomic dysfunction, however there was no statistical difference in the COMPASS-31 total scores (*p* = 0.06) or COMPASS-31 subdomain scores (Table 5). Hospitalized participants were older than non-hospitalized participants (49 ± 15.75 vs. 43 ± 17; *P* < 0.0018) and had a higher BMI (31.15 ± 13.7 vs. 26.62 ± 9.66; *P* < 0.0018). Both hospitalized and non-hospitalized participants had elevated OHQ scores [60.5 (43,77) vs. 54 (33,71.5)], elevated FSS scores [57 (47,63) vs. 53 (39,61)] and mildly elevated GAD-7 scores [8 (5, 14) vs. 8 (5, 14)]. The NPS did not differ between these two groups (*p* = 0.007). Neither group had ESS scores suggestive of excessive daytime sleepiness [9 (5, 14) vs. 8 (4, 13)]. Both hospitalized and non-hospitalized participants had relatively low scores across all RAND-36 subdomains (Table 5). The hospitalized group had lower subdomain scores on physical function (27.78 ± 33.33 vs. 44.44 ± 44.45; *p* < 0.0018), role limitations to physical health (0 ± 0 vs. 0 ± 25; *p* < 0.0018), social functioning (25 ± 46.88 vs. 37.5 ± 50; *p* < 0.0018) and general health (35 ± 30 vs. 40 ± 30, *p* < 0.0018).

**Table 5:** Comparison of demographic variables and symptoms scores in test-confirmed hospitalized and test-confirmed non-hospitalized participants.

**Variables**	**Test confirmed hospitalized (*N =* 182) *Median, IQR [25th, 75th]***	**Test confirmed non-hospitalized (*N =* 1,067)** ***Median, IQR [25*th*, 75*th*]***	**Test statistic**	**Effect size**	***p*-value**	**Comparison**
Age	49,15.75 [39.25, 55]	43,17 [35, 52]	3.76	0.11	*p < * 0.0018	CH>CNH
BMI	31.15,13.7 [24.53, 38.23]	26.62,9.66 [22.80, 32.46]	5.25	0.15	*p < * 0.0018	CH>CNH
Sex M:F (%F)	31:151 (83%)	151:928 (87%)	1.79	0.04	0.18	N/A
Symptom Duration	162,116 [91.75, 207.75]	133,137 [66, 203]	2.65	0.07	0.0078	N/A
Total COMPASS-31 Score	28.95,30.98 [15.62, 46.60]	26.4,28.35 [13.75, 42.10]	1.88	0.05	0.06	N/A
**Compass-31 Subcategories**						
Orthostatic Intolerance	16,24 [0, 24]	16,24 [0, 24]	0.91	0.03	0.36	N/A
Vasomotor	0,1.67 [0, 1.67]	0,0 [0, 0]	2.66	0.08	0.008	N/A
Secretomotor	4.29,6.43 [2.14, 8.57]	4.29,6.43 [0, 6.43]	2.57	0.07	0.01	N/A
Gastrointestinal	8.04,5.36 [5.36, 10.72]	8.04,6.25 [4.46, 10.72]	1.5	0.04	0.13	N/A
Bladder	1.11,2.22 [0, 2.22]	0,1.11 [0, 1.11]	1.98	0.06	0.05	N/A
Pupillomotor	2.33,1.58 [1.42, 3.0]	2,1.67 [1, 2.67]	2.94	0.08	0.003	N/A
Orthostatic Hypotension Questionnaire	60.5,34 [43, 77]	54,38.5 [33, 71.5]	3.77	0.11	*p < * 0.0018	CH>CNH
Fatigue Severity Scale	57,16 [47, 63]	53,22 [39, 61]	3.99	0.11	*p < * 0.0018	CH>CNH
Epworth Sleepiness Scale	9,9 [5, 14]	8,9 [4, 13]	1.72	0.05	0.09	N/A
GAD-7 Questionnaire	8,9 [5, 14]	8,9 [5, 14]	−0.09	−0.002	0.93	N/A
Neuropathic Pain Scale	24.5,50.75 [0, 50.75]	9,42 [0, 42]	2.71	0.08	0.007	N/A
**Rand-36 Subcategories**						
Physical function	27.78, 33.33 [11.11, 44.44]	44.44,44.45 [22.22, 66.67]	−6.47	−0.18	*p < * 0.0018	CNH>CH
Role limitations due to physical health	0,0 [0, 0]	0,25 [0, 25]	−3.4	−0.10	*p < * 0.0018	CNH>CH
Role limitations due to emotional problems	0,66.67 [0, 66.67]	33.33,100 [0, 100]	−2.38	−0.07	0.02	N/A
Energy/Fatigue	20,28.75 [5, 33.75]	20,30 [5, 35]	−0.69	−0.02	0.49	N/A
Emotional well-being	55,33.75 [36.25, 70]	55,32.5 [37.5, 70]	−0.42	−0.01	0.67	N/A
Social functioning	25,46.88 [3.12, 50]	37.5,50 [12.5, 62.5]	−3.56	−0.10	*p < * 0.0018	CNH>CH
Pain	45,42.5 [22.5, 65]	45,35 [32.5, 67.5]	−2.82	−0.08	0.005	N/A
General health	35,30 [20, 50]	40,30 [30, 60]	−4.25	−0.12	*p < * 0.0018	CNH>CH

We also performed a *post-hoc* analysis after excluding those with a history of autonomic dysfunction, and again found no difference in total COMPASS-31 scores between groups as the difference between groups remained non-significant [28.7 (15.2, 45.1) vs. 25.5 (13.3, 41.4) *p* < 0.08, significance threshold *p* < 0.0018].

### Correlation between COMPASS-31 and other symptom severity scales

**Figure 3:**
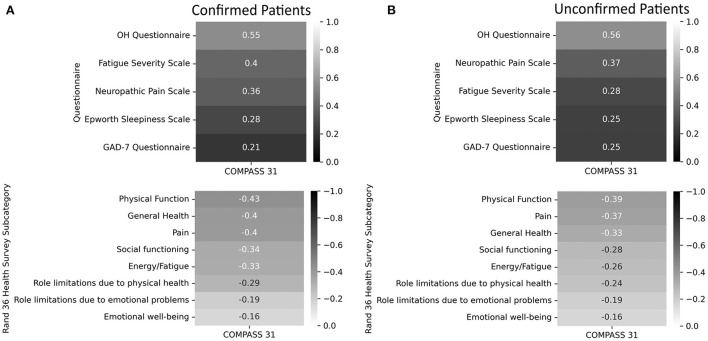
Correlations of the Fatigue Severity Scale, Neuropathic Pain Scale, Epworth Sleepiness Scale, General Anxiety Disorders Assessment, Orthostatic Hypotension Questionnaire, and the Rand-36 to total COMPASS-31 scores. A lower score on the RAND 36-Item Health Survey indicates greater disability. **(A)** Confirmed participants. **(B)** Unconfirmed participants.

The correlations between the total COMPASS-31 score and each of the other scales can be seen in Figure 3. Using a cut-off of 0.5 or −0.5, the only questionnaire with a meaningful correlation with total COMPASS-31 scores was the OHQ (test-confirmed *r* = 0.55, test-unconfirmed *r* = 0.56).

## Discussion

In this study of 2,314 individuals with PASC, we found that 66% of participants reported autonomic symptom scores consistent with moderate to severe autonomic dysfunction, suggesting a high prevalence of dysautonomia in PASC. Participants with pre-exisiting autonomic dysfunction were excluded from this prevalence estimate to avoid this potential confounding factor. Several other studies have evaluated autonomic dysfunction in PASC using the COMPASS-31. One study of 100 patients demonstrated that patients with higher COMPASS-31 scores reported greater PASC symptom burden (15). Another study of 42 patients with persistent fatigue six months after mostly mild acute infection demonstrated that 76% had COMPASS-31 scores suggestive of moderate (*n* = 21) or severe (*n* = 11) autonomic dysfunction. Our study confirms these findings in a large international cohort and supports the concept that autonomic dysfunction is highly prevalent in PASC.

Hospitalized patients in our cohort had a greater burden of orthostatic intolerance (OHQ) and fatigue (FSS), as well as worse quality of life (RAND-36), however there was no difference in total COMPASS-31 scores when compared to non-hospitalized participants (*p* = 0.06), suggesting that autonomic symptom burden in PASC is independent of the severity of the acute COVID-19 illness. In fact, the majority of our cohort were not hospitalized (88.6%), again highlighting the concept that the severity of acute COVID-19 illness is not necessarily associated with the severity of PASC.

We assessed 53 different symptoms across eight symptom domains, highlighting the heterogenous nature of PASC. The most common symptoms were fatigue, brain fog, headache, shortness of breath with exertion, body aches, palpitations, lightheadedness, and tachycardia, consistent with prior studies that have noted many of the these same symptoms in PASC patients (1, 4, 5, 16, 17). A pre-existing diagnosis of autonomic dysfunction was reported in 5.1% of test-confirmed and 8.3% of test-unconfirmed participants, with the most common autonomic disorder reported being POTS (4.1, 6.8%) at a prevalence much greater than the estimated US prevalence of 0.2–1% (18–20). However, the inclusion of those with pre-existing autonomic dysfunction did not influence our between-group comparisons of COMPASS-31 scores, thus we decided not to exclude this group to limit confounding and provide a representative cross-sectional sample of the Long-COVID community. The prevalence of pre-COVID asthma and autoimmunity reported in our cohort were also well above the rates of these medical conditions in the general US population (21, 22), raising the possibility that these medical conditions could be risk factors for developing PASC. In comparison, the prevalence of pre-COVID anxiety and depression reported in our cohort were similar to general US population rates, suggesting that anxiety and depression are not necessarily risk factors for developing PASC (23). All medical conditions analyzed except for hypertension (both groups), obesity (test-confirmed group), and current and former smoker/vaper (test-unconfirmed group) were associated with higher COMPASS-31 scores. The COMPASS-31 was originally designed to identify autonomic failure, which is associated with orthostatic hypotension and a reduction in sympathetic nervous system activity. Hypertension, obesity and tobacco use are all associated with sympathetic activation (21, 22, 24), which may be why these conditions did not correlate with elevated COMPASS-31 scores.

Test-unconfirmed participants had a substantially longer duration of symptoms compared to test-confirmed participants (*P* < 0.0018). One explanation for this is that test-unconfirmed participants were more likely to have had COVID-19 earlier in the pandemic, when access to testing and treatment was limited. Furthermore, hospitalized patients early in the pandemic would have been more likely to have been tested than those with milder disease, which might explain why participants in the test-unconfirmed group had lower rates of hospitalization (14.4 vs. 7.8%). Interestingly, the test-unconfirmed group also had significantly higher COMPASS-31 total and subdomain (except for secretomotor) scores than the test-confirmed group (*P* < 0.0018). The test-unconfirmed group also had significantly higher scores on the FSS, NPS and GAD-7, as well as lower scores on multiple subdomains of the RAND-36.

There are several possible explanations for these findings. Given that the test-unconfirmed group had COVID-19 earlier in the pandemic, participants in this group would not have had access to therapies which are now known to improve outcomes during the acute phase of the illness. In addition, those who developed COVID-19 earlier in the pandemic did not have access to COVID-19 vaccines, which have been shown to reduce the severity of COVID-19 as well as the risk of developing PASC (25). Another possibility is that test-unconfirmed participants experienced lower quality care due to lack of a unifying diagnosis and a discounting of poorly understood symptoms by clinicians, resulting in delayed access to treatment. Lastly, it is possible that some of the test-unconfirmed participants did not actually have COVID, however this would not explain why the test-unconfirmed group had higher COMPASS-31 scores. It is worth noting that 63.6 % of the test-unconfirmed participants were diagnosed with COVID-19 by a clinician.

The elevated OHQ and FSS scores seen in our cohort are consistent with prior studies which have demonstrated that orthostatic intolerance syndromes (26–30) and fatigue (1, 5, 16, 17) are very common in PASC. Interestingly, none of the groups had excessively high ESS scores, suggesting that fatigue is more common than sleepiness in PASC. The mildly elevated GAD-7 scores seen in all groups is in line with data demonstrating an increase in anxiety in the general population during the pandemic (23). All groups reported substantial impairment across all subdomains of the RAND-36, highlighting the significant impact that PASC has on quality of life, which is particularly profound in the domains of “role limitations to physical health” and “role limitations to emotional problems.”

Of all the questionnaires evaluated in this study, the only questionnaire that was strongly correlated with COMPASS-31 scores was the OHQ. It is not surprising that the OHQ scores correlated with higher COMPASS-31 scores considering that disorders of orthostatic intolerance such as POTS and OH are among the most common autonomic disorders seen in PASC (4). The FSS, ESS, and GAD-7 were likely not strongly associated with autonomic dysfunction because fatigue, sleepiness, and anxiety can occur, but are not specific to autonomic dysfunction.

We were surprised that the RAND-36 subdomains did not have a stronger correlation with COMPASS-31 scores and expected that the presence of autonomic dysfunction in PASC would have had a greater impact on quality of life. However, these findings do not exclude the possibility that autonomic dysfunction in PASC is associated with a reduced quality of life, but rather suggest that PASC, even in the absence of autonomic dysfunction, has a very high burden of disability.

There were limitations to our study. The study population was limited to those who were English speakers and had access to the PASC support groups and social media channels. This may have also limited the diversity of our cohort, as most our participants were white, further limiting the generalizability of our findings to minority groups and communities of color. Given that this was a survey study and variables were self-reported, information bias and misclassification could affect the validity of the results. In addition, this study lacked a control group and baseline measures in participants. Also, because the study participants were recruited from long-COVID-19 support groups, our findings may not be generalizable to all PASC patients. Thus, selection bias may have influenced our results, and caution should be taken when generalizing these study findings to the entire PASC population. Another limitation is that the COMPASS-31 was designed to assess autonomic failure and is less specific in assessing other forms of autonomic dysfunction. Thus, it is possible that some of the symptoms screened for in the COMPASS-31 could be explained by COVID effects on end-organs rather than primary autonomic dysfunction. Finally, lack of confirmed-positive COVID test results in all patients represents a limitation inherent to the design of our survey-based study, however this is counterbalanced by the benefits of utilizing an online survey to access a large, global population of PASC patients, as well as the opportunity to include those infected early in the pandemic without access to COVID testing.

In conclusion, this is the largest study to date that has utilized validated autonomic questionnaire scores to demonstrate that autonomic dysfunction is highly prevalent in PASC. COVID severity did not correlate with the severity of autonomic dysfunction in our cohort, suggesting that even mild cases of COVID can result in significant autonomic symptom burden. Given the role of the autonomic nervous system in regulating immune function and coagulation pathways, both of which appear to play a role in PASC (31, 32), future studies should focus on mechanisms of autonomic dysfunction, their relationship to immune and coagulation biomarkers, and potential interventions that can improve autonomic function. Finally, clinicians should be aware of the prevalence of autonomic impairment in those with PASC. Patient-completed COMPASS-31 surveys and clinician-administered bedside orthostatic stand testing may be helpful screening tools to identify PACS patients with autonomic dysfunction.

## Data availability statement

The raw data supporting the conclusions of this article will be made available by the authors, without undue reservation.

## Ethics statement

The studies involving human participants were reviewed and approved by Stanford University and Stony Brook University Institutional Review Boards. The patients/participants provided their written informed consent to participate in this study.

## Author contributions

MM, LES, SM, and NL conceived the project and designed the survey. MM and LES recruited the survey participants. CT and RS accessed and edited the raw data. RS and NL analyzed the quantitative data. RS and LS performed the statistical analyses. NL, RS, and LS created the tables and figures. NL drafted the manuscript, with revisions by MM, LS, SM, LG, HB, and LES. All authors contributed to the article and approved the submitted version.

## Funding

Open access publication fee was funded by Dysautonomia International.

## Conflict of interest

Author NL—Grant from Dysautonomia International; Stock or stock options in Health Care Select SPDR, Ishares Biotechnology. Author LS—Consulting fees from Eisai Pharmaceuticals (ad board, unrelated to current manuscript) and Jazz Pharmaceuticals (ad board, unrelated to current manuscript); Payment or honoraria for lectures, presentations, speakers bureaus, manuscript writing or educational events from Jazz Pharmaceuticals, Xywav, Xyrem and Sunosi; Payment for expert testimony from City and County of San Francisco (sleep deprivation cases); Support for attending meetings and/or travel from Jazz Pharmaceuticals (Psych Congress 2021, poster presentation); Leadership or fiduciary role in other board, society, committee or advocacy group from Graduate Education Subcommittee Chair (AAN, unpaid), Graduate Education Subcommittee Chair (AAN, unpaid), Editorial Board (Practical Neurology, unpaid); Stock or stock options in Alphabet. Author SM—Grants or contracts from the Myasthenia Gravis Foundation of America; Consulting fees from Argenx, Alexion and UCB pharma (consultation and advisory board meetings). Author LG—Consulting fees from United Health Group. Author MM—Royalties from Elsvier Inc.; Consulting fees from 2nd MD, Infinite MD and Guidepoint LLC; Payments for CME lectures from MED-IQ; Payment for expert testimony from Van Cott Talamante (expert witness); Medical Advisory Board of Dysautonomia International (unpaid), and MSA Coalitionn (unpaid).

The remaining authors declare that the research was conducted in the absence of any commercial or financial relationships that could be construed as a potential conflict of interest.

## Publisher's note

All claims expressed in this article are solely those of the authors and do not necessarily represent those of their affiliated organizations, or those of the publisher, the editors and the reviewers. Any product that may be evaluated in this article, or claim that may be made by its manufacturer, is not guaranteed or endorsed by the publisher.
